# Challenging Parenting Behaviour and Anxiety Disorders in Emerging Adulthood

**DOI:** 10.1007/s10826-022-02434-7

**Published:** 2023-02-15

**Authors:** Evie Wai Ting Chan, Kelly Tsz Ching Wong, Laura H. Clark

**Affiliations:** 1 Department of Psychology, Durham University, Durham, UK; 2 Department of Psychology, Northumbria University, Newcastle, UK

**Keywords:** Challenging parenting behaviour, Anxiety disorder, Cognitive avoidance, Intolerance of uncertainty, Emerging adulthood

## Abstract

Challenging parenting behaviour (CPB) refers to parental encouragement of behaviours where children push their own limits through engaging them engage in safe risks, such as rough-and-tumble play (Bögels & Phares, 2008). Preliminary evidence suggests that CPB reduces the risk of child anxiety however, little is known about the relationship between CPB and specific forms of anxiety disorders and the factors that influence this relationship. The present study aims to examine current maternal and paternal CPB in relation to symptoms of generalised anxiety disorder (GAD) and social anxiety disorder (SAD) in emerging adulthood, and to identify whether intolerance of uncertainty (IU) and cognitive avoidance (CA) sequentially mediate this relationship. A sample of 190 UK-based adults (aged 18–25) completed a battery of online self-report measures. Greater maternal CPB, but not paternal CPB, was found to predict lower symptoms of SAD, but not GAD. IU and CA did not sequentially mediate the relationship between CPB and symptoms of GAD or SAD. This study suggests that CPB may be associated with certain forms of anxiety disorders such as SAD, but further investigation is needed to understand the mechanisms between CPB and anxiety in young people.

## Highlights

- Challenging parenting behaviour was found to have an impact on anxiety disorders in emerging adulthood.
- Relative to mothers, fathers engaged in more challenging parenting behaviour towards their emerging adult offspring.
- Emerging adults perceived fathers to challenge them more in competitions, while mothers were more likely to encourage an engagement in novelty.
- Greater maternal overall challenging parenting behaviour and paternal encouragement of social assertiveness predicted reduced symptoms of social anxiety.
- Intolerance of uncertainty and cognitive avoidance were individually associated with symptoms of anxiety disorders, yet did not mediate their relationships with challenging parenting behaviour.

Anxiety disorders are one of the most common diagnoses in mental health, with a predicted global prevalence of 7.8% for young adults aged 18–34 years (Baxter et al., 2013). Childhood and adolescent anxiety disorders are associated with academic underachievement, poor socio-occupational outcomes, and other psychopathologies at aged 30 (Essau et al., 2014). In order to develop effective early interventions and treatments, an in-depth understanding of anxiety disorders is necessary. Thus far, a range of risk factors for anxiety has been proposed, including adverse life events, genetics, and parental behaviours (see Murray et al., 2009, for an integrative review). Although the role of parental factors in anxiety has been extensively researched (e.g. Yap et al., 2014), it is only recently that the concept of challenging parenting behaviour (CPB) has been introduced (Bögels & Phares, 2008; Majdandžić et al., 2016).

The term CPB refers to the parental encouragement of children to engage in physical and other socioemotional challenges that push them beyond their comfort zone for the purposes of fostering competence, promoting confidence, and reducing the risk of anxiety (Bögels & Phares, 2008; Majdandžić et al., 2016; Paquette, 2004). According to Bögels and Phares (2008), CPB provides children with the opportunity to explore the external environment and to handle different challenges, with consideration of the child’s competencies and safety. Examples of CPB include rough-and-tumble play, teasing, the encouragement of assertion and risk-taking, and exposure to defeats. Parental engagement in and the effects of CPB may differ according to parental sex; Paquette (2004) has previously characterised father-child relationships as one of activation, in which children are empowered to be open to the outside world by encouraging them to explore the environment and take safe chances. While fathers tend to spend less time with their children (Geary, 2010), their engagement in physical play is usually greater than in mother-child interactions (Clarke-Stewart, 1978; Möller et al., 2013). At present however, the majority of anxiety research predominantly focuses on mothers in spite of the evidence for differences in maternal and paternal parenting behaviours. The importance of considering the complex interactions between each member of a family unit has been further emphasised by Cabrera et al. (2018). Therefore, we aim to contribute to the extant paternal literature by considering both the independent and the additive influences of maternal and paternal CPB on child development.

Literature suggests that the differing impact of maternal and paternal CPB may vary according to age. Möller et al. (2015) administered questionnaires to parents in Amsterdam and reported infant anxiety at 10–15 months to be significantly related to lower levels of paternal CPB, with no link to maternal CPB. Parental sex differences in CPB have also been demonstrated in toddlers through observational methods (Majdandžić et al., 2014). The research team investigated whether CPB at baseline would predict changes in the social behavioural inhibition of siblings aged 2 and 4, 6-months later. Paternal and maternal CPB only emerged as significant predictors for the 4-year-olds, with the former as a negative and the latter a positive predictor of child social behavioural inhibition. Taken together, both paternal and maternal CPB may be related to social anxiety in older children. Furthermore, research has provided evidence of a negative association between paternal CPB and anxiety symptoms in children beyond infancy and toddlerhood. For instance, Lazarus et al. (2016) presented a negative association between maternal and paternal CPB and symptoms of anxiety in Australian children (aged 3–4) based on parental reports and maternal diagnostic interviews. A longitudinal study in the Netherlands has also found both maternal and paternal CPB to negatively predict child anxiety symptoms (Majdandžić et al., 2018). It is important to note that in this study, CPB was measured based on observations both at home and in the laboratory at 1 and 2.5 years, whilst parents rated child anxiety symptoms at 2.5 and 4.5 years. Thus, both questionnaire and observational methodologies have provided support for the buffering role of CPB for anxiety symptoms in early childhood.

Despite the gathering momentum of literature on CPB, there is limited empirical literature on CPB and specific forms of anxiety in emerging adulthood (i.e. between the ages of 18–25). This period may carry important mental health implications as it is characterised by identity exploration and marked changes in academic, professional, and social domains (Arnett, 2000). It is estimated that 9.6% of males and 26.7% of females report having an anxiety disorder between the ages of 19 and 36 years (Gustavson et al., 2018). Amongst the most prevalent anxiety disorders in youth are generalised anxiety disorder (GAD) and social anxiety disorder (SAD), with an estimated global lifetime prevalence of 3.7% and 4.0%, respectively (Ruscio et al., 2017; Stein et al., 2017). According to the DSM-V (American Psychiatric Association, 2013), SAD is characterised by a persistent fear or anxiety towards social situations due to the potential of embarrassment, negative evaluation, and scrutiny by others, often leading to extensive or subtle avoidance behaviour. GAD is characterised by Further research is needed to investigate CPB in relation to specific anxiety disorders, such as GAD and SAD, during emerging adulthood.

To our knowledge, one study to date has examined current CPB and young adult anxiety (Smout et al., 2020). In a sample of Australians aged 18–25, Smout et al. (2020) developed and validated a new CPB measure: the Challenging Parenting Behaviour Questionnaire Emerging Adult Version (CPBQ-EA). Three subscales were established: social assertiveness (i.e. social CPB), competitiveness (i.e. competition-based CPB), and novelty attempts (i.e. novelty-based CPB). While there was no parental sex difference in social CPB, young adults did report higher paternal engagement in competition-based CPB which is consistent with the proposed traditional role of fathers in physical play (Paquette, 2004). Participants also reported a greater level of maternal novelty-based CPB that may be attributed to mothers having a greater awareness of the novel opportunities offered to their children. In line with this, mothers have been found to share a more open and better quality communication with their child and have a better understanding about the events occurring in a young adult’s life (Rosnati et al., 2007; Nelson et al., 2011). Given that both constructs were based on social domains, it is perhaps not surprising that paternal social CPB significantly predicted lower social anxiety (as measured by the Social Interaction Anxiety Scale [SIAS]). No other CPB subscales by either parent was a significant predictor of anxiety but it is important to note that this study used a general measure of anxiety symptoms (i.e the anxiety scale of the Depression, Anxiety and Stress Scale – 21 Items [DASSas]) rather than a specific measure of GAD. Furthermore, the DASSas only shares a weak-to-moderate correlation with the widely recognised GAD-screening tool, Generalised Anxiety Disorder-7 scale (GAD-7; Rutter & Brown, 2017). Consequently, in order to investigate CPB in relation to symptoms of GAD and SAD in emerging adulthood, the use of a measure specific to these disorders is needed.

While CPB may buffer the development of child anxiety, the specific mechanisms through which this occur have yet to be explored. Existing theoretical frameworks suggest that one potential mediator may be child cognition (e.g. Wood et al., 2003). Intolerance of uncertainty (IU) is a cognitive schema that refers to the dispositional tendency to respond negatively to uncertainty due to its perceived association with negative outcomes, regardless of the likelihood of their occurrence (Carleton, 2016; Dugas et al., 2004). The CBT-IU protocol targets IU through situational exposure such that individuals can review their core negative beliefs about uncertainty and evaluate their coping ability even in the occurrence of a negative outcome (Dugas & Robichaud, 2007; Robichaud, 2013). In a similar way, CPB may allow children the opportunity to develop their self-efficacy and to test the belief that uncertainty would always lead to disastrous consequences, thereby increasing their tolerance to uncertainty and reducing their risk of clinical anxiety. Supporting this, IU has been found as a partial mediator in the relationship between parental overprotection and trait anxiety in university students in Pakistan (Zafar & Jami, 2016). A study of American university students has also found IU to mediate the association between current self-reported anxiety and retrospective reports of parental anxious rearing, as characterised by excessive worry about the child’s safety and well-being (Zlomke & Young, 2009). Although some studies have suggested IU to be unique to GAD (e.g. Ladouceur et al., 2000), it has also been linked to the severity of social anxiety (Boelen & Rijntjes, 2009). Therefore, IU may predispose individuals to clinical levels of social and generalised anxiety and may be an important transdiagnostic mechanism in understanding the relationship between CPB and these forms of anxiety disorders in emerging adulthood.

Cognitive avoidance (CA) is another well-established transdiagnostic mechanism associated with anxiety disorders (e.g. Dickson et al., 2012) which may also be important when trying to understand the influence of IU on anxiety. CA reflects the cognitive enforcement of disengagement coping strategies in order to disrupt and to avoid stressful, unwanted mental contents (Borkovec et al., 2004; Koerner & Dugas, 2006). These strategies include distraction, thought suppression (i.e. the conscious elimination of threatening thoughts), and thought substitution (i.e. the replacement of distressing thoughts for neutral or positive thoughts). By promoting threat perception even for ambiguous situations, individuals high in IU may employ CA strategies to prepare for and to manage the distress associated with those situations (Dugas et al., 1998). Paradoxically however, CA may impede the extinguishing of anxiety responses instead (Newman & Llera, 2011); an association between CA and symptom severity has been found for patients with GAD (Dugas et al., 2007). Therefore, CPB may reduce the risk of anxiety by reducing IU tendency while IU may indirectly foster anxiety through CA. Although CA has been suggested to be more characteristic of GAD than of SAD (e.g. Hearn et al., 2017), CA has been found to mediate the path from IU to somatic anxiety (i.e. a physical manifestation of anxiety common in SAD) in Italian undergraduates (Botessi et al., 2016). Given the lack ofstudies on IU and CA in relation to CPB and anxiety disorders, it would be of particular interest to examine this relationship in an emerging adult population.

Despite the importance of disorder-specific research in this area, there is a lack of empirical investigations aimed at understanding specific anxiety disorders in relation to emerging adult CPB. Advancing current understanding in this area may not only provide further support for the theoretical conceptualisation of CPB, but would also allow us to better target specific forms of clinical anxiety in emerging adulthood. Specifically, we aimed to investigate the relationship between maternal and paternal CPB and symptoms of SAD and GAD, as reported by young adults. A secondary aim was to identify if IU and CA sequentially mediate the path from CPB to symptoms of SAD and GAD. With consideration of Smout et al.’s (2020) findings, we hypothesised (1) a greater level of total paternal CPB, as compared to total maternal CPB, would be reported by participants (2) a greater level of paternal competition-based CPB than maternal, a greater level of maternal novelty-based CPB than paternal, and similar levels of paternal and maternal social CPB would be reported (3) total paternal CPB, and individual subscales of paternal CPB (i.e. social assertiveness, competitiveness, and novelty), would negatively predict symptoms of SAD and GAD (4) that total maternal CPB, and individual subscales of maternal CPB (i.e. social assertiveness, competitiveness, and novelty) would negatively predict symptoms of GAD and SAD; (5) that the relationship between paternal CPB and symptoms of GAD and SAD would be sequentially mediated by IU and CA; and (6) that the relationship between maternal CPB and symptoms of GAD and SAD would be sequentially mediated by IU and CA.

## Method

### Participants

Participants aged 18–25 years based in the United Kingdom were recruited from either social media or the participant pool for Durham University psychology students. Seven participants were excluded because they either failed to meet the age requirement or had withdrawn from the study. Demographic information did not differ between participants who had completed the study and those who did not. The final sample included 190 participants (*M* = 20.59, SD = 1.81), of whom 167 (87.9%) were female. The majority of the sample was Caucasian (*n* = 140; 74.7%) undergraduate students (*n* = 146; 76.8%). Ninety percent of participants came from two-parent (opposite sex) families (*n* = 171). Further descriptive summaries for each demographic variable are available in Table 1.

**Table 1:** Descriptive statistics for demographic variables (*n* = 190)

Demographic variable	Frequency	Percentage (%)
Age (mean, SD)	20.6	1.8
Gender		
Female	167	87.9
Male	22	11.6
Other	1	0.5
Ethnicity		
Caucasian	140	74.7
Asian	34	17.9
Other	15	7.9
Prefer not to say	1	0.5
Education		
GCSEs or Equivalent	1	0.5
A-Levels or Equivalent	12	6.3
Undergraduate	146	76.8
Postgraduate	21	11.1
Doctorate/PhD	7	3.7
Other	3	1.6
Occupation		
Student	169	88.9
Part-time employment	6	3.2
Full-time employment	14	7.4
Prefer not to say	1	0.5
Family Composition^a^		
Two-parent (different sex)	171	90.0
Two-parent (same sex)	1	0.5
Sole parent (mother)	12	6.3
Sole parent (father)	2	1.1
Other	4	2.1

^a^Reflects predominant family composition before the age of 18

### Measures

The Challenging Parenting Behaviour Questionnaire Emerging Adult Version (CPBQ-EA; Smout et al., 2020) is a measure of current engagement in CPB as perceived by young adults. It was adapted from the original parent-report measure of CPBQ (Majdandžić et al., 2010). The CPBQ-EA contains 20 statements designed to capture the perception of the respondent on both maternal and paternal use of CPB on a five-point Likert scale, from 1 (not applicable) to 5 (completely applicable). Separate versions of the CPBQ-EA were completed for fathers and mothers. The CPBQ-EA consisted of three subscales: social (10 items; e.g. ‘my mother/father encourages me to speak my mind and back myself’), competition-based (5 items; e.g. ‘my mother/father tries to beat me at sports’), and novelty-based CPB (5 items; e.g. ‘my mother/father encourages me to talk to new people and pursue new interests’). Total scores range from 20 to 100, with higher scores indicating a more frequent usage of CPB. Smout et al. (2020) found a good to excellent internal consistency for CPBQ-EA subscales in a non-clinical sample for both the father (α = 0.85–0.93) and mother version (α = 0.82–0.93). In the present study, total CPB demonstrated strong internal consistency for both the father (α = 0.95) and mother (α = 0.92) version. The CPBQ-EA subscales for fathers (social: α = 0.95; competition-based: α = 0.81; novelty-based: α = 0.93) and mothers (social: α = 0.92; competition-based: α = 0.80; novelty-based: α = 0.90) also revealed good-to-excellent reliability.

The English version of the Intolerance of Uncertainty Scale (IUS; Buhr & Dugas, 2002) is a 27-item scale that assesses individuals’ beliefs and responses to uncertain events (e.g. that uncertainty is unacceptable and/or can lead to stress or frustration; Dugas et al., 2004). Respondents rate the extent to which they agree with statements relating to uncertainty (e.g. ‘Uncertainty stops me from having a firm opinion’) on a five-point Likert scale from 1 (not at all characteristic of me) to 5 (entirely characteristic of me). The total score ranges from 27 to 135, with a higher score indicating a greater level of IU. The English-translated version of IUS has been found to have excellent internal consistency (α = 0.94) and a good test-retest reliability of 0.74 (Buhr & Dugas, 2002). For the present study, the IUS was found to have strong internal consistency with a Cronbach’s alpha of 0.96.

The English version of the Cognitive Avoidance Questionnaire (CAQ; Sexton & Dugas, 2008) is a 25-item self-report measure that was translated from the original French version (Gosselin et al., 2002). It assesses the tendency to engage in CA strategies (e.g. ‘I think about things that concern me as if they were occurring to someone else’). Each item is rated on a five-point Likert scale from 1 (not at all typical) to 5 (completely typical). Scores are summed from 25 to 125, with higher scores indicating greater levels of CA. The CAQ has demonstrated strong internal consistency (α = 0.95) and test-retest reliability (*r* = 0.85) for undergraduates (Sexton & Dugas, 2008). The internal reliability of CAQ in the present study was excellent (α = 0.95).

The Generalised Anxiety Disorder 7-item (GAD-7; Spitzer et al., 2006) is a brief 7-item measure that assesses the symptom severity of GAD according to the DSM-IV diagnostic criteria (American Psychiatric Association, 2000). Participants report the frequency at which they experience symptoms of GAD (e.g. ‘Feeling nervous, anxious or on edge’) on a four-point Likert scale from 0 (not at all) to 3 (nearly every day). Scores ranged from 0 to 21, where higher scores represented a higher frequency of GAD symptoms. Strong internal consistency and test-retest reliability were found in both clinical (Kroenke et al., 2007) and community samples (Löwe et al., 2008). In the present study, an excellent internal consistency was observed (α = 0.89).

The Social Interaction Anxiety Scale (SIAS; Mattick & Clarke, 1998) is comprised of 20 items that measure symptoms of social interaction anxiety. Respondents rate items on a five-point Likert scale, with the total score ranging from 0 to 80. A higher score indicates a higher level of social interaction anxiety. Three positively worded items (item number 5, 9, and 11) require reverse scoring; all others are negatively worded. The scale has been shown to have strong internal consistency and test-retest reliability in clinical, community, and undergraduate samples (α = 0.88–0.94), as well as convergent and divergent validity (Heimberg et al., 1992; Mattick & Clarke, 1998). In the current study, the internal reliability of SIAS was excellent (α = 0.94).

### Procedures

The study was approved by the Durham University Psychology Department Human Ethics Sub-Committee. Participants were invited to complete an online survey through a study advertisement posted on a university student page on a social media platform (Facebook) and in the Durham University undergraduate psychology participant pool. Participants recruited from the participant pool received course credit for their participation, but no incentives were used for participants recruited through social media. The study invite contained a link to an online survey hosted on Qualtrics, an online research software. Participants were presented with the study information sheet, online informed consent sheet, a demographic questionnaire, the questionnaire measures described above, and then a debrief sheet. Informed consent was gained online from participants prior to the study. The father and mother versions of the CPBQ-EA, as well as GAD-7 and SIAS, were presented in a randomised order. The study took an average of 37.61 min to complete.

### Data Preparation

A priori power calculation was undertaken for mediation analysis. Assuming a medium effect size (*R*^2^ = 0.15) and a significance level of α = 0.05 with four predictor variables, 85 participants were required to achieve 80% power. Data was exported to Statistical Package for Social Science (SPSS) 26.0. The distribution of all measures violated the Shapiro–Wilk and Kolmogorov–Smirnov significance statistics (*p* < 0.05). Both paternal and maternal total CPB scores and CAQ scores indicated considerable positive skewness (skewness > 1), while IUS and SIAS demonstrated negative skewness (skewness < −1). Standardised scores (*z*) did not exceed 3.29, implying the absence of a univariate outlier (Tabachnick & Fidell, 2001). Square-root, logarithmic, and reciprocal transformations failed to improve distributions. Non-parametric test equivalents were therefore used to analyse the original data.

Spearmen’s Rho correlation, Kruskal–Wallis, and Mann–Whitney U tests were used for descriptive analysis involving continuous or categorical demographic variables. Differences in maternal and paternal CPB were examined with Wilcoxon signed-rank tests (hypotheses 1 and 2). Hierarchical multiple regression analyses (MRA) using bootstrapping with 1000 replications were conducted for CPB scores on symptoms of anxiety; a separate hierarchical MRA was carried out for GAD (hypothesis 3) and SAD (hypothesis 4). Due to the theoretical relevance of CPB to fathers (e.g. Bögels & Phares, 2008), paternal CPB was entered in step 1 and maternal CPB entered in step 2. The hierarchical MRAs were then repeated with the order reversed. There was no evidence of multicollinearity for any variables; all tolerance and VIF values were within the accepted standard of >0.01 and <10, respectively (Field, 2013). The serial mediating effects of IU and CA in the relationship between CPB and symptoms of anxiety were examined using Hayes’ PROCESS macro (model six) with 5000 bootstrap samples (Hayes, 2018). Separate mediation analysis was conducted for paternal and maternal CPB, and GAD and SIAS.

## Results

### Preliminary Analyses

A series of Mann–Whitney U tests showed significantly higher IU scores for females (mean rank = 97.7) than for males (mean rank = 70.6), *U* = 2352, *z* = 2.19, *p* = 0.028. Likewise, females (mean rank = 97.4) expressed significantly greater symptoms of GAD than males (mean rank = 63.1), *U* = 2372, *z* = 2.76, *p* = 0.006. Using Spearman’s rank order correlations, age was significantly associated with lower paternal total CPB, *r*_s_(188) = −0.15, *p* = 0.039, and with lower paternal novelty-based CPB, *r*_s_(188) = −0.16, *p* = 0.029.

A series of Kruskal–Wallis H tests were conducted to examine scale variables in relation to categorical demographic data. Distributions for paternal total CPB were significantly different between groups of family composition, *X*^2^(4) = 14.36, *p* = 0.006. There was also a significant difference in distributions across family compositions for paternal social CPB, *X*^2^(4) = 18.22, *p* = 0.001, as well as for paternal competition-based CPB, *X*^2^(4) = 11.95, *p* = 0.018. Subsequent pairwise comparisons with a Bonferroni correction indicated a significant difference in paternal total CPB between two-parent (opposite sex) households (mean rank = 100.4) and single-parent (mother) households (mean rank = 45.5), *p* = 0.001. Likewise, there was a significant difference in paternal social CPB between a two-parent (opposite sex) household (mean rank = 100.9) and single-parent (mother) household (mean rank = 35.0), *p* = 0.001. Paternal competition-based CPB was significantly different between two-parent (opposite sex) households (mean rank = 99.9) and single-parent (mother) households (mean rank = 57.8), *p* = 0.010. However, neither gender or family composition were controlled for in the main analysis as nearly 90% of our sample was female and from a two-parent (opposite sex) household.

Descriptive statistics and correlation coefficients of the relationship between each scale variable are displayed in Table 2. Maternal and paternal total CPB and its subscales all shared a significant positive correlation (*p* < 0.005). While there was a significant negative association between maternal total CPB and IUS (*p* < 0.05), the latter was significantly related to increased CAQ (*p* < 0.001). Symptoms of GAD were associated with lower level of paternal novelty-based CPB and maternal total, social, and novelty-based CPB (*p* < 0.05), as well as significantly greater IUS, CAQ, and SIAS scores (*p* < 0.001). Scores of SIAS were associated with lower reported paternal social CPB and maternal total CPB and its subscales, as well as significantly greater IUS and CAQ (*p* < 0.05). There was also a significant positive relationship in SIAS with both IU and CA (*p* < 0.001).

**Table 2:** The relationships between each scale variable (*n* = 190)

Variable	*M*	SD	1	2	3	4	5	6	7	8	9	10	11
Paternal CPBQ-EA													
1. Total CPBQ-EA	64.5	19.8	—										
2. Social CPBQ-EA	36.1	11.3	0.93^**^	—									
3. Competition-based CPBQ-EA	12.5	5.1	0.64^**^	0.41^**^	—								
4. Novelty-based CPBQ-EA	15.9	6.3	0.91^**^	0.80^**^	0.46^**^	—							
Maternal CPBQ-EA													
5. Total CPBQ-EA	64.7	15.8	0.56^**^	0.54^**^	0.30^**^	0.54^**^	—						
6. Social CPBQ-EA	37.4	9.4	0.55^**^	0.58^**^	0.26^**^	0.47^**^	0.91^**^	—					
7. Competition-based CPBQ-EA	9.7	4.7	0.34^**^	0.29^**^	0.29^**^	0.33^**^	0.56^**^	0.31^**^	—				
8. Novelty-based CPBQ-EA	17.5	5.4	0.44^**^	0.40^**^	0.20^**^	0.49^**^	0.83^**^	0.70^**^	0.25^**^	—			
9. IUS	73.2	23.1	−0.03	−0.09	0.10	−0.02	−0.12^*^	−0.12	−0.11	−0.07	—		
10. CAQ	72.0	21.0	0.08	0.03	0.11	0.09	−0.07	−0.09	0.05	−0.07	0.66^**^	—	
11. GAD-7	9.5	5.6	−0.09	−0.12	0.05	−0.12^*^	−0.16^*^	−0.12^*^	−0.12	−0.17^**^	0.68^**^	0.58^**^	—
12. SIAS	36.1	17.9	−0.10	−0.18^**^	0.07	−0.08	−0.25^**^	−0.28^**^	−0.13^*^	−0.19^**^	0.61^**^	0.46^**^	0.52^**^

^*^p < 0.05; ^**^ *p* < 0.01

### Differences in Paternal and Maternal Challenging Parenting Behaviour

Given the absence of symmetry in the distribution of differences for maternal and paternal total CPB, a sign test was used to evaluate the differences between paternal and maternal CPB (hypotheses 1 and 2). The median of paternal total CPB (68) was higher than that of maternal total CPB (66), *z* = −2.06, *p* = 0.040, which supports hypothesis 1. The median of paternal competition-based CPB (13) was also higher than that of maternal competition-based CPB (9), *z* = −5.48, *p* < 0.001. In contrast, the median of novelty-based CPB was higher for mothers (19) than for fathers (16), *z* = 2.41, *p* = 0.016. There was no difference in the medians of social CPB between fathers (38.5) and mothers (39), *z* = 0.08, *p* = 0.938. Therefore, support is also provided for hypothesis 2 as well.

### Challenging Parenting Behaviour and Generalised Anxiety Disorder

Model statistics of CPB as predictors of GAD are presented in Table 3. For model 1 which considered paternal and maternal total CPB (hypotheses 3 and 4), there was an independence of residuals given the Durbin-Watson statistic of 1.84. The full model was not statistically significant, *R*^2^ = 0.02, *F*(2, 184) = 1.81, *p* = 0.166. Model fit was not significantly improved by the addition of maternal total CPB in step 2, ∆*R*^2^ = 0.01, ∆*F*(1, 184) = 2.12, *p* = 0.147. Symptoms of GAD were not significantly predicted by either paternal or maternal total CPB in step 1 or 2, whether or not gender and family composition were controlled for. Maternal total CPB reached statistical significance in step 1 when it was entered first, *B* = −0.06, *b* = −0.17, 95% CI [−0.11, −0.01], *p* = 0.020, but not without controlling for gender and family composition (*p* = 0.059). While these findings do not support the hypothesised role of total paternal CPB in GAD (hypothesis 3), partial support is provided for hypothesis 4 regarding maternal total CPB.

**Table 3:** Hierarchical multiple linear regression: Paternal and maternal CPB as predictors of symptoms of anxiety disorders

Variable	*Symptoms of GAD*					*Symptoms of SAD*				
	*B*	*β*	BCa 95% CI		*p*	*B*	*β*	BCa 95% CI		*p*
			*LL*	*UL*				*LL*	*UL*	
Model 1										
*Step 1*										
Paternal Total CPBQ-EA	−0.03	−0.09	−0.07	0.02	0.213	−0.10	−0.11	−0.22	0.04	0.166
*Step 2*										
Paternal Total CPBQ-EA	−0.01	−0.02	−0.05	0.05	0.830	0.03	0.04	−0.12	0.20	0.695
Maternal Total CPBQ-EA	−0.05	−0.13	−0.11	0.02	0.135	−0.29	−0.26	−0.48	−0.10	0.005
Model 2										
*Step 1*										
Paternal Social CPBQ-EA	−0.01	−0.02	−0.13	0.11	0.855	−0.40	−0.26	−0.72	−0.03	0.021
Paternal Competition-based CPBQ-EA	0.14	0.13	−0.05	0.32	0.122	0.50	0.14	−0.05	1.10	0.070
Paternal Novelty-based CPBQ-EA	−0.15	−0.17	−0.36	0.08	0.208	0.14	0.05	−0.57	0.81	0.687
*Step 2*										
Paternal Social CPBQ-EA	−0.03	−0.06	−0.17	0.10	0.659	−0.28	−0.17	−0.63	0.10	0.137
Paternal Competition-based CPBQ-EA	0.15	0.14	−0.04	0.33	0.116	0.54	0.15	−0.05	1.11	0.071
Paternal Novelty-based CPBQ-EA	−0.07	−0.07	−0.31	0.20	0.586	0.31	0.11	−0.46	1.05	0.429
Maternal Social CPBQ-EA	0.05	0.08	−0.12	0.21	0.590	−0.35	−0.18	−0.83	0.11	0.132
Maternal Competition-based CPBQ-EA	−0.06	−0.05	−0.28	0.14	0.593	−0.20	−0.05	−0.83	0.40	0.526
Maternal Novelty-based CPBQ-EA	−0.19	−0.18	−0.41	0.05	0.110	−0.19	−0.06	−0.92	0.70	0.596

*B* unstandardised bootstrapped regression coefficient, *β* standardised regression coefficient, *BCa 95% CI* bias-corrected and accelerated 95% bootstrap interval, *LL* lower limit, *UL* upper limit

When paternal and maternal CPB subscales were considered in model 2 (hypotheses 3 and 4), the Durbin-Watson statistic (1.83) indicated an independence of residuals. The full model was not statistically significant, *R*^2^ = 0.05, *F*(6, 180) = 1.41, *p* = 0.213. The addition of maternal CPB subscales to paternal CPB did not significantly improve model fit, ∆*R*^2^ = 0.02, ∆*F*(3, 180) = 1.05, *p* = 0.372. Neither paternal nor maternal subscales were significant predictors of symptoms of GAD in the model at either step. Maternal CPB subscales remained non-significant predictors even if entered first in step 1, and controlling for gender and family composition revealed similar results. Consequently, neither hypothesis 3 nor 4 was supported by the current data.

#### Paternal CPB and GAD: the mediating role of IU and CA

**Fig. 1:**
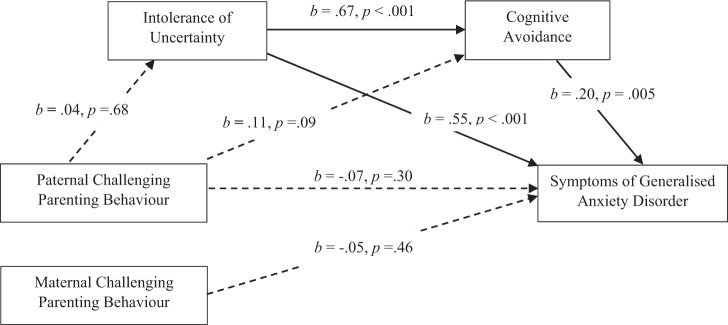
Mediation Model of IU and CA in the Relationship Between Paternal CPB and Symptoms of GAD. Total effect: *b* = −0.01, SE = 0.02, 95% CI [−0.05, 0.04]*. β* = −0.02. Direct effect: *b* = −0.02, SE = 0.02, 95% CI [−0.05, 0.02], *β* = −0.07. Total indirect effect: *b* = −0.01, SE = 0.02, 95% CI [−0.02, 0.05], *β* = 0.05. Mediation model assessed using Hayes’ process model six (2018) evaluating IU and CA as mediators of the relationship between paternal CPB and symptoms of GAD. Significant pathways are denoted by solid arrowed lines; non-significant pathways are denoted by dotted arrowed lines. *b* = unstandardised regression coefficient. *β* = completely standardised regression coefficient of the indirect effect. CI = bias-corrected and bootstrapped confidence intervals based on 5000 samples

Figure 1 shows the mediation model of IU and CA in the relationship between paternal CPB and GAD (hypothesis 5). The total effect of the model, the direct effect of paternal CPB, and the overall indirect effect were not significant. Paternal CPB did not significantly affect symptoms of GAD through IU, *b* = 0.01, SE = 0.01, 95% CI [−0.02, 0.03], or through CA, *b* = 0.01, SE = 0.01, 95% CI [−0.002, 0.02]. Neither was the indirect serial mediation effect through IU and CA significant, *b* = 0.001, SE = 0.004, 95% CI [−0.01, 0.01], providing no support for hypothesis 5. All of the effects remained non-significant even when gender and family composition were added as covariates.

#### Maternal CPB and GAD: the mediating role of IU and CA

**Fig. 2:**
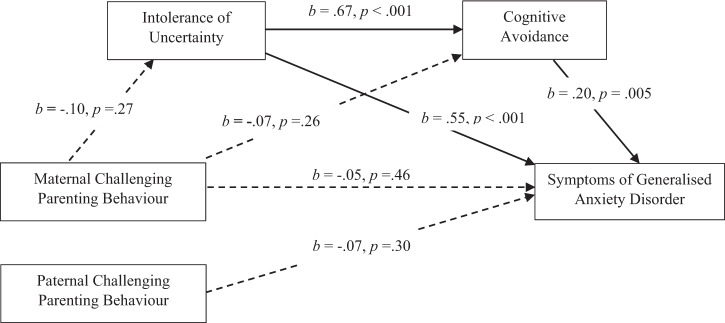
Mediation Model of IU and CA in the Relationship Between Maternal CPB and Symptoms of GAD. Total effect: *b* = −0.05, 95% CI [−11, 0.02], *β* = −0.13. Direct effect: *b* = −0.02, 95% CI [−0.06, 0.03], *β* = −0.05. Total indirect effect: *b* = −0.03, 95% CI [−0.07, 0.01], *β* = −0.08. Mediation model assessed using Hayes’ process model six (2018) evaluating IU and CA as mediators of the relationship between maternal CPB and symptoms of GAD. Significant pathways are denoted by solid arrowed lines; non-significant pathways are denoted by dotted arrowed lines. *b* = unstandardised regression coefficient. *β* = completely standardised regression coefficient of the indirect effect. CI = bias-corrected and bootstrapped confidence intervals based on 5000 samples

The mediation analysis of IU and CA in maternal CPB and GAD (hypothesis 6) is presented in Fig. 2. The analysis showed a non-significant total effect as well as non-significant direct and overall indirect effects. The indirect effect of maternal CPB on symptoms of GAD was non-significant, whether through IU, *b* = −0.02, SE = 0.02, 95% CI [−0.05, 0.01], CA, *b* = −0.01, SE = 0.01, 95% CI [−0.02, 0.004], or IU and CA, serially, *b* = −0.01, SE = 0.01, 95% CI [−0.02, 0.003]. Controlling for gender and family composition did not change the pattern of results, and so hypothesis 6 was not supported in the analysis.

### Challenging Parenting Behaviour and Social Anxiety Disorder

Model statistics of CPB as a predictor of symptoms of SAD (hypotheses 3 and 4) are shown in Table 3. An independence of residuals was evidenced by the Durbin-Watson statistic of 2.08. The full model was statistically significant, *R*^2^ = 0.06, *F*(2, 184) = 5.45, *p* = 0.005. Model fit was significantly improved by the addition of maternal total CPB, ∆*R*^2^ = 0.05, ∆*F*(1, 184) = 8.76, *p* = 0.003. While paternal total CPB was not a significant predictor in either step, maternal total CPB was a significant negative predictor when considered alone in step 1, *B* = −0.26, *b* = −0.23, 95% CI [−0.42, −0.13], *p* < 0.001, and in the final model, *B* = −0.28, *b* = −0.25, 95% CI [−0.44, −0.15], *p* = 0.002. This pattern of results persisted even with family composition and gender controlled for. Therefore, hypothesis 4 but not 3 was supported by the current analysis.

In the second model of CPB subscales (hypotheses 3 and 4), there was an independence of residuals as shown by the Durbin-Watson statistic (1.99). The full model was statistically significant, *R*^2^ = 0.09, *F*(6, 180) = 2.81, *p* = 0.012. Maternal CPB subscales provided a significant improvement to the model fit, ∆*R*^2^ = 0.04, ∆*F*(3, 180) = 3.78, *p* = 0.042. Symptoms of SAD were not significantly predicted by any maternal or paternal CPB subscales. When maternal CPB subscales were entered first instead, maternal social CPB reached statistical significance as a negative predictor of SAD in step 1, *B* = −0.44, *b* = −0.23, 95% CI [−0.87, −0.04], *p* = 0.042. This continued to be the case when family composition and gender were controlled for. Controlling for these covariates also led paternal social CPB to become a significant negative predictor of SIAS scores, *B* = −0.48, *b* = −0.30, 95% CI [−0.92, −0.10], *p* = 0.018, though only in step 1 when maternal CPB subscales were not considered as well. These findings partially support hypotheses 3 and 4 of the current study.

#### Paternal CPB and SAD: the mediating role of IU and CA

**Fig. 3:**
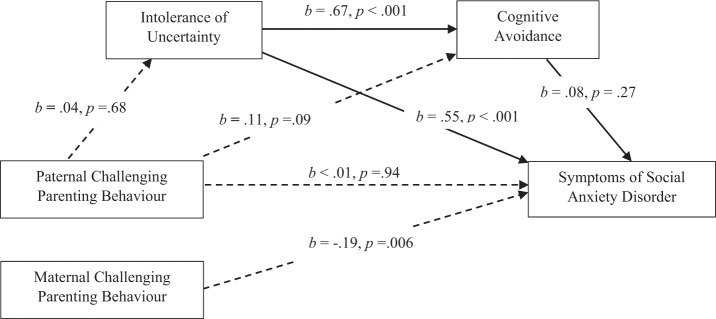
Mediation Model of IU and CA in the Relationship Between Paternal CPB and Symptoms of SAD. Total effect: *b* = 0.03, 95% CI [−0.12, 0.19], *β* = 0.04. Direct effect: *b* = 0.004, 95% CI [−0.12, 0.13], *β* = 0.005. Total indirect effect: *b* = −0.03, 95% CI [−0.07, 0.13], *β* = 0.03. Mediation model assessed using Hayes’ process model six (2018) evaluating IU and CA as mediators of the relationship between paternal CPB and symptoms of SAD. Significant pathways are denoted by solid arrowed lines; non-significant pathways are denoted by dotted arrowed lines. *b* = unstandardised regression coefficient. *β* = completely standardised regression coefficient of the indirect effect. CI = bias-corrected and bootstrapped confidence intervals based on 5000 samples

Figure 3 displays the mediating roles of IU and CA in paternal total CPB and SIAS scores (hypothesis 5). There was a non-significant total effect and direct effect. Neither did paternal CPB significantly influence symptoms of SAD through IU, *b* = 0.02, SE = 0.05, 95% CI [−0.08, 0.11], CA, *b* = 0.01, SE = 0.01, 95% CI [−0.01, 0.03], or serially through IU and CA, *b* = 0.002, SE = 0.01, 95% CI [−0.01, 0.02]. The overall indirect effect was not significant, with effects remaining non-significant when family composition and gender were added as covariates. Consequently, hypothesis 5 was not supported.

#### Maternal CPB and SAD: the mediating role of IU and CA

**Fig. 4:**
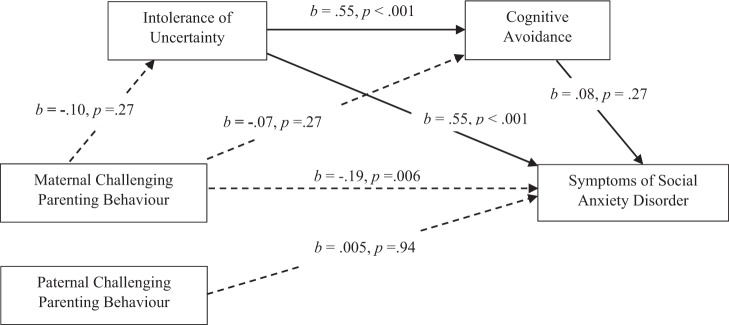
Mediation Model of IU and CA in the Relationship Between Maternal CPB and Symptoms of SAD. Total effect: *b* = −0.29, 95% CI [−0.48, −0.10], *β* = −0.25. Direct effect: *b* = −0.21, 95% CI [−0.36, −0.06], *β* = −0.19. Total indirect effect: *b* = −0.07, 95% CI [−0.18, 0.04], *β* = −0.07. Mediation model assessed using Hayes’ process model six (2018) evaluating IU and CA as mediators of the relationship between maternal CPB and symptoms of SAD. Significant pathways are denoted by solid arrowed lines; non-significant pathways are denoted by dotted arrowed lines. *b* = unstandardised regression coefficient. *β* = completely standardised regression coefficient of the indirect effect. CI = bias-corrected and bootstrapped confidence intervals based on 5000 samples

Figure 4 presents the mediation analysis for maternal CPB and symptoms of SAD (hypothesis 6). Data indicated significant total and direct effects, but a non-significant overall indirect effect. Neither IU, *b* = −0.06, SE = 0.05, 95% CI [−0.17, 0.04], or CA, *b* = −0.01, SE = 0.01, 95% CI [−0.04, 0.01], were significant independent mediators in the relationship between maternal CPB and symptoms of SAD. Nor was the serial mediating effect of IU and CA significant, *b* = −0.01, SE = 0.01, 95% CI [−0.03, 0.01]. The total standardised indirect effect accounted for 28% of the standardised total effect of maternal CPB on social anxiety. The addition of family composition and gender as covariates yielded a similar pattern of results, and therefore no support was shown for hypothesis 6.

## Discussion

In the current study, we aimed to investigate the relationships between CPB, IU, CA, and symptoms of GAD and SAD, as reported by a sample of emerging adults. This is the first study to examine IU and CA as potential mediators of the relationship between CPB and anxiety in young adults, and also the first to look into disorder-specific anxiety symptoms. Moreover, the current study not only investigated CPB as a broad concept but also as specific forms of the construct. Although maternal CPB was found to predict reduced symptoms of SAD, the serial mediating effect of IU and CA in the relationship between CPB, and GAD and SAD was not significant. Participants reported a greater level of overall paternal CPB than maternal CPB, as well as a more frequent use of competition-based CPB from their fathers and more novelty-based CPB from their mothers.

In the current investigation, greater overall maternal CPB predicted fewer SAD symptoms in young adults even when paternal CPB, gender, and household differences were controlled for. This partially supports the notion that CPB may prevent anxiety symptoms but is inconsistent with studies that have found only paternal CPB as a significant predictor of child anxiety (e.g. Lazarus et al., 2016; Majdandžić et al., 2018). This suggests that perhaps the impact of paternal and maternal CPB vary by the age of the child. Although paternal social CPB and maternal total CPB were found to predict symptoms of SAD and GAD, respectively, these findings became non-significant after the relevant CPB scale(s) of the other parent was entered into the regression model. The identified shared variance between paternal and maternal CPB may support existing research that maternal and paternal parenting are interdependent and interrelated (e.g. Barnett et al., 2008; Li et al., 2022). While we did not find any maternal CPB subcomponents to predict symptoms of SAD or GAD, the combined effect of maternal encouragement of social assertiveness, competitiveness, and novelty did predict fewer SAD symptoms. These findings highlight the importance of further research into how subdomains of CPB interact as well as how CPB may be unique to SAD. There are two potential explanations for the specificity of CPB to SAD; the most likely explanation is that emerging adulthood involves major social transition during which young adults are increasingly exposed to social novelty outside of the family, and so CPB may be particularly important to reducing symptoms of social anxiety in this period. The second explanation may be based on social modelling (Bandura & Walters, 1977), which suggests that children tend to learn and imitate what parents encourage them to do. Thus, findings indicate that the importance of CPB may vary according to the age of the child, the parental gender, and the form of anxiety disorder.

The present study did not find evidence that the pathway between parental CPB (both maternal and paternal) and symptoms of GAD or SAD is sequentially mediated by IU and CA, or by either alone. In regards to CPB and IU specifically, a recent insight proposed that the stress response to uncertainty is a default response that is normally under tonic prefrontal inhibition when one’s safety is perceived (Brosschot et al., 2016). This default response is now believed to be developed before birth rather than learnt in response to a specific environment (Brosschot et al., 2018). This research might, at least in part, explain why IU was found to be less influenced by parenting behaviours in the same way that other factors that are at least partially genetically determined have been shown by research to predispose disorders, such as personality traits (Krueger et al., 2008).

Although IU did not mediate the relationship between CPB and symptoms of anxiety disorders, it was found to predict symptoms of SAD and GAD. These findings support existing research that individuals who are intolerant of uncertainty may be prone to experiencing SAD and GAD symptoms, providing further support of IU as a transdiagnostic mechanism of these disorders (Boelen & Rijntjes, 2009; Dugas et al., 2004; Grad, 2011). IU was also found to be positively associated with CA, which supports the theory that individuals with higher IU may attempt to engage in CA strategies in order to reduce uncertainty-related distress (Dugas et al., 1998). Findings concerning CA strategies also reveal it to be positively associated with symptoms of GAD and SAD, supporting the perpetuation of one’s anxiety by this avoidant coping response, or vice versa (Newman & Llera, 2011). In sum, these findings outline the significance of IU and CA in the symptoms of GAD and SAD in young adults.

The paths from IU to symptoms of SAD and GAD via CA were also non-significant, despite IU and CA both acting as independent correlates of GAD and SAD. These findings stand in contrast to other research that has found CA to mediate the relationship between IU and somatic anxiety (Bottesi et al., 2016), which may reflect differences in mechanisms across anxiety disorders and/or age differences. While IU was suggested to be innate, CA may be learned and varies across different ages. Theurel and Gentaz (2018) reported that older adolescents used less distraction and more rumination strategies. This finding may explain why CA failed to mediate the relationship between IU and the two types of anxiety here – it may be more affected by factors other than IU. Future research is required to examine alternative cognitive mechanisms that may explain the relationship between CPB and anxiety. Given that CPB involves parental encouragement of activities that safely push children beyond their limits, this type of parenting may build a child or young person’s confidence and competence in undertaking information processing and problem-solving tasks (Bögels & Phares, 2008; Majdandžić et al., 2014), and in turn, reduce the risk of anxiety. Therefore, future research might consider investigating CPB in relation to cognitive competence (which can refer to cognitive processes such as creative thinking and critical thinking skills including self-regulation) as it relates to one’s ability to internalise and manipulate cognitive processes in order to interpret the environment and to guide their behaviour (Shek et al., 2013).

A greater frequency of paternal CPB was reported relative to maternal CPB, which supports that overall, fathers may be more likely to emphasise “exploration to outside world” than mothers (Paquette, 2004). This finding supports and extends previous self-report and observational findings of greater levels of paternal CPB than maternal for children (e.g. Lazarus et al., 2016; Majdandžić et al., 2014) in young adults. In line with existing theories and findings on the roles of fathers and mothers in rearing children (Smout et al., 2020), fathers were also reported to exhibit more competition-based CPB than mothers. This finding suggests that fathers do indeed tend to interact with their child more physically, and are more likely to stimulate engagement in risk-taking behaviours than mothers (Paquette, 2004). Also consistent with Smout et al.’s (2020) study, participants reported a higher frequency of novelty-based CPB in mothers than in fathers, but similar levels of encouragement in social assertiveness. Nelson et al. (2011) suggested that mothers are better informed about events in young adults’ life than fathers while parental care and overprotection may reduce as children grows (Tillery et al., 2015). Consequently, mothers may be more likely to encourage their children to engage in novel situations. Alternatively, the lack of differences in social CPB between fathers and mothers may be due to the low sensitivity of the CPBQ-EA social subscale in capturing differences between their encouragement of social assertiveness. Previous literature illustrated that fathers and mothers both promote child social competence and assertiveness but in different ways, as fathers serve a challenging and reassuring role while mothers function as a regulator (Leidy et al., 2013). Such differencee in parental presentation of CPB may not have been detected through the subscale items. Moreover, the significantly higher paternal social and competition-based CPB in two-parent households relative to in single-mother households suggests that single mothers may not take up a stereotypical parental role in rearing children. This finding also indicates that fathers and mothers may each use CPB in a very different and distinct manner. Thus, our findings demonstrate that both fathers and mothers play a role in CPB with different usage of CPB, and how they use CPB may vary across the offspring’s developmental stages.

A number of limitations should be acknowledged. Firstly, the correlational, cross-sectional design of our study limits the extent to which causality and directionality can be inferred in the relationships between scale variables. Secondly, our sample has an over-representation of females (87.9%) and two-parent (opposite sex) families (90.0%), limiting the generalisability of the findings. Thirdly, the use of self-report measures may have undermined the validity of results as responses were subject to personal perception and biases (Gaylord-Harden et al., 2003), and the perspectives of other informants (e.g. parents) should be incorporated in the future. Fourthly, this study focused only on current CPB, without measuring or controlling for a retrospective perception of parenting (i.e. parenting experienced at a younger age). Finally, data collection was conducted during the first wave of Coronavirus (COVID-19); reports of anxiety symptoms may therefore have been affected by the pandemic and the lockdown policies imposed in the United Kingdom. Future research should aim to explore other cognitive factors that may influence the relationship between CPB and anxiety (e.g. cognitive competence). Researchers may also review the CPBQ-EA subscales in mapping them with the theoretical basis of CPB. Most importantly, research should investigate the specificity of SAD in relation to CPB, and also examine CPB in relation to other forms of anxiety.

In conclusion, the present study contributes to literature on CPB in relation to two specific forms of anxiety disorders: GAD and SAD. Our findings provide a better understanding on the relationship between CPB, GAD and SAD in young adulthood. The findings from the current study suggest a relationship between CPB and symptoms of social anxiety in emerging adulthood. The study findings indicate that evaluating parenting style, in particular components of CPB, may be important when evaluating and treating symptoms of SAD in young people. This finding may also suggest the importance of evaluating parenting interventions (e.g. parental psychoeducational material) that promote the use of CPB in young people with symptoms of SAD in future research. However, the relationship between CPB and symptoms of anxiety could not be explained by IU and CA and other transdiagnostic mechanisms should be investigated. Additionally, although greater rates of competition-based CPB were reported in fathers, mothers were indicated to encourage young adults to engage in novelty more frequently, indicating that both parents may play a role in CPB but in different ways.
